# Mouse Models of Intracerebral Hemorrhage in Ventricle, Cortex, and Hippocampus by Injections of Autologous Blood or Collagenase

**DOI:** 10.1371/journal.pone.0097423

**Published:** 2014-05-15

**Authors:** Wei Zhu, Yufeng Gao, Che-Feng Chang, Jie-ru Wan, Shan-shan Zhu, Jian Wang

**Affiliations:** 1 Department of Emergency Medicine, Tongji Hospital, Tongji Medical College, Huazhong University of Science and Technology, Wuhan, Hubei, PR China; 2 Department of Anesthesiology and Critical Care Medicine, Johns Hopkins University, School of Medicine, Baltimore, Maryland, United States of America; 3 Department of Biological Sciences, Illinois Institute of Technology, College of Science, Chicago, Illinois, United States of America; 4 Department of Psychiatry and Behavioral Sciences, Johns Hopkins University, School of Medicine, Baltimore, Maryland, United States of America; Indian Institute of Integrative Medicine, India

## Abstract

Intracerebral hemorrhage (ICH) is a devastating condition. Existing preclinical ICH models focus largely on striatum but neglect other brain areas such as ventricle, cortex, and hippocampus. Clinically, however, hemorrhagic strokes do occur in these other brain regions. In this study, we established mouse hemorrhagic models that utilize stereotactic injections of autologous whole blood or collagenase to produce ventricular, cortical, and hippocampal injury. We validated and characterized these models by histology, immunohistochemistry, and neurobehavioral tests. In the intraventricular hemorrhage (IVH) model, C57BL/6 mice that received unilateral ventricular injections of whole blood demonstrated bilateral ventricular hematomas, ventricular enlargement, and brain edema in the ipsilateral cortex and basal ganglia at 72 h. Unilateral injections of collagenase (150 U/ml) caused reproducible hematomas and brain edema in the frontal cortex in the cortical ICH (c-ICH) model and in the hippocampus in the hippocampal ICH (h-ICH) model. Immunostaining revealed cellular inflammation and neuronal death in the periventricular regions in the IVH brain and in the perihematomal regions in the c-ICH and h-ICH brains. Locomotor abnormalities measured with a 24-point scoring system were present in all three models, especially on days 1, 3, and 7 post-ICH. Locomotor deficits measured by the wire-hanging test were present in models of IVH and c-ICH, but not h-ICH. Interestingly, mice in the c-ICH model demonstrated emotional abnormality, as measured by the tail suspension test and forced swim test, whereas h-ICH mice exhibited memory abnormality, as measured by the novel object recognition test. All three ICH models generated reproducible brain damage, brain edema, inflammation, and consistent locomotor deficits. Additionally, the c-ICH model produced emotional deficits and the h-ICH model produced cognitive deficits. These three models closely mimic human ICH and should be useful for investigating the pathophysiology of ICH in ventricle, cortex, and hippocampus and for evaluating potential therapeutic strategies.

## Introduction

Intracerebral hemorrhage (ICH), one of the most damaging types of stroke, is associated with high morbidity and mortality [1], [2]. ICH most commonly affects the basal ganglia, but it can often occur in lobar regions, cerebellum, and brainstem sites [3]. In recent years, much progress has been made in our understanding of ICH-induced brain injury. The initial bleed and the physiologic response to hematoma cause the primary brain injury, and inflammation contributes to the progression of secondary brain injury [4]–[7]. One characteristic of brain inflammation is microglial activation [8], which occurs much earlier than neutrophil infiltration and acts as a major cellular mediator of secondary brain injury after ICH [4], [7], [9]. Astrocytes are also activated after ICH [4], [5]. Despite these recent findings, the exact mechanisms of ICH-induced brain injury are still not fully known.

Animal models that accurately mimic the complexity and heterogeneity of ICH in humans are essential for systematically studying the pathophysiology and treatment of ICH. Some of the methods used to model ICH in rodents and large animals include mechanical balloon inflation, thrombin, collagenase, and donor or autologous blood injection [5], [10]. Rodents are most often used to model ICH [5], [11]. Over the past decades, rat has been the most frequently used species for preclinical ICH studies [5]. However recently, mice have become favored for determining the cellular and molecular mechanisms of injury after ICH because of the growing applications of transgenic and knockout animals.

Two models are primarily used to mimic clinical ICH in mice [4], [11], [12]. One is a collagenase model, in which injected collagenase causes vessel rupture and induces intracerebral bleeding. The second is the autologous whole blood model, in which injected blood is used to study blood toxicity. The collagenase-induced ICH model was first introduced by Rosenberg and colleagues in rats in 1990 [13]. We established and characterized this ICH model in mice in 2003 [14]. We and others went on to establish the double autologous whole blood ICH model in mice [15], [16]. Since then, we have used these two controllable, reliable, and reproducible ICH models to investigate the pathophysiology of striatal ICH and to evaluate potential therapeutic targets [4]. However, both models have limitations and mimic only some features of clinical ICH [4], [11], [12].

To date, preclinical models of ICH have targeted striatum. Few animal studies have modeled ICH by targeting other sites, such as ventricle, cortex, and hippocampus. In fact, the cortex is the second most common brain region affected by ICH in humans [3], and damage to the frontal cortex causes emotional problems. Furthermore, intraventricular hemorrhage (IVH), which occurs from extension of thalamic and ganglionic bleeding into the ventricular space, is associated with high mortality. Hippocampal injury can also be debilitating because the hippocampus is necessary for memory formation [17] and damage to it can cause cognitive impairment. Behavioral tests are able to detect post-stroke functional deficits and are useful for studying functional recovery after ICH [5]. Most behavioral tests in rodents have been used to evaluate locomotor function after striatal ICH. Few tests evaluate damage to cortex and hippocampus by assessing emotional and cognitive impairment.

Recently, researchers developed ICH models in rats by injecting autologous blood into the ventricle [18], [19] or hippocampus [20]. Additionally, a cortical ICH (c-ICH) model was established in mouse by stereotactic injection of collagenase into the right cortex [21]. Based on our extensive experience with striatal ICH models in mice [14], [22]–[24], we have established protocols for mouse models of IVH, c-ICH, and hippocampal ICH (h-ICH). We validated and characterized the ICH models by assessing ventricular volume, hematoma volume, brain edema, neuroinflammation, neuronal death, and neurobehavioral deficits, including those related to motor function, emotion, and cognition.

## Materials and Methods

### Animals

This study was carried out with male C57BL/6 mice (18–25 g, total n = 112) in strict accordance with the recommendations in the Guide for the Care and Use of Laboratory Animals from the National Institutes of Health. The animal protocol was approved by the Johns Hopkins University Animal Care and Use Committee (Approved protocol number: MO12M111). Animals were housed under controlled laboratory conditions (12-h light/dark cycle, controlled temperature and humidity, ad lib access to food and water). All efforts were made to minimize the number of mice used and any potential suffering.

### Surgical procedure and experimental protocol

Mice were placed into a clean induction chamber and anesthetized by isoflurane (3–4% for induction and 1–2% for maintenance, Baxter Healthcare Co. Deerfield, IL) evaporated in an oxygen-air mixture (20%:80%). Throughout the experimental period, rectal temperature was maintained at 37±0.5°C by an electronic thermostat-controlled warming blanket (Stoelting Co., Wood Dale, IL). Body temperature was maintained until animals completely recovered from anesthesia and displayed normal motor activity. After animals were returned to their home cages, they were monitored closely over the next 4 h and then daily for the rest of the study.

For the IVH model, we randomly selected mice (n = 23) and placed them in a stereotaxic frame (Stoelting Co.) under isoflurane anesthesia. A 1-cm-long midline incision was made in the scalp, beginning midway between the eyes and terminating behind the lambda. A cotton swab was used to clear away the soft tissue covering the skull. A Hamilton syringe was mounted onto the injection pump and the needle positioned directly over the bregma. The x, y, and z axis coordinates were all set to zero. The needle was then positioned at the entry point, 0.5 mm posterior and 1.0 mm lateral of the bregma to the right. A small cranial burr hole was drilled through the skull at the entry point. The mouse's tail was disinfected with 70% alcohol and immersed in warm, sterile water (40°C) for 2 min. After puncturing the central tail artery with a sterile needle (25 G), we collected blood onto a piece of Parafilm (Pechiney Plastic Packaging Co., Chicago, IL, USA) and then drew 25 µL into the Hamilton syringe. The needle was slowly inserted into the right ventricle to a depth of 2.5 mm below the surface of the skull, and the blood was injected at a rate of 5 µL/min. The needle was left in place for 10 min and then removed at a rate of 1 mm/min to prevent the reflux of blood. The burr hole was filled with bone wax (Ethicon, Somerville, NJ), and the scalp incision was closed with cyanoacrylate glue (Henkel Consumer Adhesive Inc. Scottsdale, Arizona). Sham control mice (n = 17) were injected with an equal volume of saline.

For the c-ICH and h-ICH models, we used injections of collagenase VII-S (sterile-filtered, high purity, Sigma-Aldrich, St. Louis, MO) dissolved in saline. Mice (n = 19) were randomly selected and placed in a stereotaxic frame as in the IVH model. For the c-ICH model, we injected 0.4 µL of collagenase (150 U/mL) into two injection sites of the right frontal cortex (Bregma coordinates: 0.0 mm anterior and 1.5 mm lateral and 1.0 mm anterior and 2.0 mm lateral; 1.6 mm ventral) [21]. The needle was left in place for 20 min after the first injection; then the second site was injected and the needle left in place for another 20 min. For the h-ICH model, we injected 0.2 µL of collagenase into the CA1 region of the right hippocampus (Bregma coordinates: 2.5 mm posterior, 1.7 mm lateral, and 1.8 mm ventral) [25]. The needle was left in place for 10 min before removal. Needles were removed from all injection sites at a rate of 1 mm/min. An equal volume of saline was injected into each site of sham control mice (n = 17). For detailed, step-by-step instructions that describe each model, see Text S1.

### Hemorrhagic injury volume and ventricular volume

On day 3 after ICH, five mice in each group were anesthetized and perfused intracardially with 4% paraformaldehyde in 0.1 mol/L phosphate-buffered saline (pH 7.4). The brains were removed, kept in 4% paraformaldehyde for 24 h, and then immersed in 30% sucrose for 72 h at 4°C. The entire brain of each mouse was cut into 50-μm-thick sections with a cryostat. All sections except for those from the IVH group were stained with Luxol fast blue (for myelin) and Cresyl violet (for Nissl bodies). Sections from the IVH group were stained only with Cresyl violet. SigmaScan Pro software (version 5.0.0 for Windows; Systat, Port Richmond, CA) was used to quantify volumes of the ventricle and lesion. The volume of the ventricles in cubic millimeters was calculated according to a published method [26]. The volume of the lesion in cubic millimeters was calculated by multiplying the thickness by the sum of the damaged areas of each section [14]. Sections were analyzed by an investigator blinded to the experimental cohort.

### Brain water content

Brain edema was determined in all mice by the wet–dry weight ratio method as described previously [27]. Briefly, mice (n = 4/group) were sacrificed by decapitation 72 h post-ICH. The brains were removed immediately and cut from 1 mm anterior of bregma to 3 mm posterior of bregma in the IVH group, from 3 mm anterior of bregma to 2 mm posterior of bregma in the c-ICH group, or from 0.8 mm posterior of bregma to 4.0 mm posterior of bregma in the h-ICH group. In the IVH and c-ICH groups, the brains were dissected into five parts: ipsilateral and contralateral basal ganglia, ipsilateral and contralateral cortex, and cerebellum. In the h-ICH group, the brains were dissected into ipsilateral and contralateral hippocampus, ipsilateral and contralateral cortex, and cerebellum. In all groups, the cerebellum served as an internal control. Brain samples were weighed immediately on an analytical balance (Denver Instrument Co., Bohemia, NY) to obtain the wet weight and then dried at 100°C for 48 h to obtain the dry weight. Brain edema was expressed as (wet weight − dry weight)/wet weight of brain tissue × 100%.

### Immunofluorescence

Immunofluorescence was carried out as described previously [27]. Sections (n = 5 mice/group) were incubated with rabbit anti-Iba 1 (microglial marker; 1∶500; Wako Chemicals, Richmond, VA), rabbit anti-glial fibrillary acidic protein (GFAP, astrocyte marker; 1∶500; Dako, Carpinteria, CA), and rabbit anti-myeloperoxidase (MPO, neutrophil marker; 1∶500; Dako). Stained sections were examined with a fluorescence microscope (Eclipse TE2000-E, Nikon, Tokyo, Japan). We averaged the numbers of immunoreactive cells over a 40× microscopic field from 12 randomly selected locations per mouse (4 fields per section × 3 sections per mouse) in the periventricular brain region (IVH model) or in the perihematomal brain regions (c-ICH and h-ICH models). We express the results as positive cells per square millimeter. Sections were analyzed by an investigator blinded to the experimental cohort.

### Histology

Fluoro-Jade B (FJB) staining was used to quantify neuronal death (n = 4 mice/group) as described previously [27]. Stained brain sections were examined with a fluorescence microscope (Eclipse TE2000-E). The number of FJB-positive cells was averaged over a 20× microscopic field from 12 randomly selected locations per mouse (4 fields per section × 3 sections per mouse) in the periventricular brain region (IVH model) or in the perihematomal brain regions (c-ICH and h-ICH models). We express the results as positive cells per square millimeter. Sections were analyzed by an investigator blinded to the experimental cohort.

### Behavioral tests

Mice were housed in a temperature- and humidity-controlled room that was maintained on a 12-h light/dark cycle. All behavioral tests were conducted during the light cycle phase in an enclosed behavior room. The same animals were used for testing motor, memory, and emotional behavior. All behavioral tests were evaluated and analyzed by an investigator blinded to the groups.

#### Neurologic deficit score

We tested each mouse for neurologic deficits on days 1, 3, 7, 14, and 21 post-ICH. Using a modified protocol that we have published [28], we scored mice on six parameters, including body symmetry, gait, climbing, circling behavior, front limb symmetry, and compulsory circling. Each test was graded from 0 to 4, establishing a maximum deficit score of 24.

#### Wire-hanging test

We used a previously published wire-hanging test with minor modification to evaluate grip strength, balance, and endurance in mice on days 1, 3, 7, 14, and 21 post-ICH [29], [30]. An iron wire (1 mm in diameter, 55 cm long) was stretched horizontally between two posts, 50 cm above the ground. Mice were placed on the wire and had to suspend their body weight with their forelimbs. To prevent the mice from using all four paws, we gently covered their hind limbs with adhesive tape. A pillow was placed beneath the mice to prevent injury from falls. The time that the mouse was able to remain suspended was recorded.

#### Tail suspension test (TST)

The TST was performed in each mouse on day 21 post-ICH. According to methods described previously [31], [32], mice were suspended by their tails at the edge of a shelf 55 cm above a desk. Adhesive tape (17 cm long) was used to stick the tail (approximately 1 cm from the tip) to the shelf. A plastic tube (4 cm long, 1 cm in diameter, 1.5 g) around the tail prevented the mouse from climbing its tail. A camera recorded the movement of the mouse for 6 min, and the duration of mobility was recorded during this period. The immobility time was calculated by subtracting the total amount of mobility time from the 360 s of test time. Lack of escape-related behavior was considered immobility. Mice that hung passively and completely motionless were considered immobile.

#### Forced swim test (FST)

On day 22 post-ICH, each mouse underwent the FST based on an established protocol with minor modifications [33], [34]. Mice were placed individually in cylindrical tanks (20 cm high, 22 cm in diameter) containing 10 cm of water at 24±1°C. A camera recorded the movement of mice for 6 min. The immobility time was calculated by subtracting the total amount of mobility time during the last 4 min from the 240 s of test time. The mouse was judged to be immobile when it ceased struggling and remained floating motionless in an upright position in the water, making only small movements to keep its head above the water [35].

#### Novel object recognition test

On day 21 post-ICH, we used an established protocol [36] to test each mouse in the novel object recognition test. On the first day, the mouse was placed in a cage (47×26×20 cm) and habituated to exploring an open field for 5 min. On the second day, two identical novel objects (green cubes, 4×4×3 cm) were placed in the arena, and the mouse was allowed to explore them for 10 min. After 1 h, the mouse was given one novel object (white ball, 5 cm in diameter) and one familiar one (green cube) and allowed to explore for 5 min. A camera recorded the behaviors displayed by each mouse during the test. We compared the total time spent exploring the new and old objects. Time spent exploring an object included time in direct contact and time within the object area. The mouse was considered to be in the object area if its nose was directed at the object and less than approximately 2 cm from it. A discrimination index (total time spent with new object/total time devoted to exploration of objects) was calculated for each animal.

### Statistical analysis

All data are expressed as mean ± SD. Differences between two groups (sham vs. model group) were analyzed with independent sample t-tests. For behavioral tests, data at different time points for the same group were compared by one-way ANOVA followed by the Bonferroni post hoc test, and data for different groups at the same time point were compared by two-way ANOVA followed by the Bonferroni post hoc test. A *p* value <0.05 was considered statistically significant.

## Results

The mortality of mice was 3 of 23 (13.0%) in the IVH group, 1 of 19 (6.7%) in the c-ICH group, and 1 of 19 (6.7%) in the h-ICH group. No animals died in any of the sham groups (0 of 51).

### Histologic morphology

**Figure 1:**
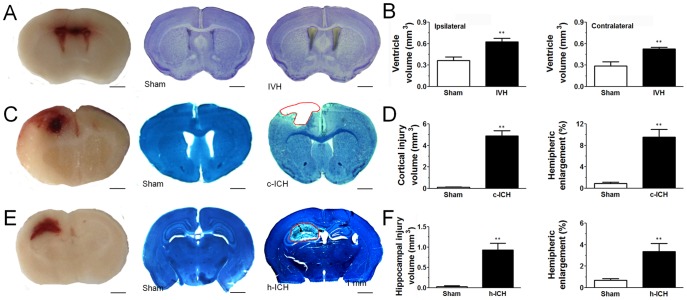
Brain injuries produced by intraventricular hemorrhage (IVH), cortical intracerebral hemorrhage (c-ICH), and hippocampal ICH (h-ICH). (A) Photographs of frozen brain sections 72 h after infusion of 25 µL autologous whole blood into the ventricle. Left: Accumulated blood can be seen in bilateral ventricles. Middle and right: Sections from sham (middle) and IVH mice (right) stained with Cresyl violet. (B) Quantification showed that both ipsilateral and contralateral ventricular volumes were greater in IVH mice than in sham mice (n = 5 mice per group). (C) Representative unstained brain sections and brain sections stained with Luxol fast blue/Cresyl violet obtained 72 h after sham procedure or c-ICH. Hematoma was present in the frontal cortex in the c-ICH group. (D) Quantification showed significantly larger injury volume and hemispheric enlargement in the c-ICH group than in the sham group (n = 9 mice per group). (E) Representative unstained brain sections and brain sections stained with Luxol fast blue/Cresyl violet obtained 72 h after the sham procedure or h-ICH. Hematoma was present in the hippocampus in the h-ICH group. (F) Quantification showed significantly larger injury volume and hemispheric enlargement in the h-ICH group than in the sham group (n = 5 mice per group). Scale bar = 1 mm. Values are means ± SD; **p<0.01, t-test.

In the IVH group, accumulated blood was observed in both ventricles at 24 h (data not shown) and 72 h after infusion of autologous blood into the right ventricle. Additionally, Cresyl violet staining revealed enlargement of the ventricles 72 h after ICH (n = 5 mice/group, *p*<0.01 vs. control; Fig. 1A and 1B). Although the degree of ventricular dilatation was greater on the side ipsilateral to infusion, the difference between the sides was not statistically significant (*p*>0.05; Fig. 1B).

At 72 h after collagenase injection, hematoma was present in the cortex in the c-ICH group and in the hippocampus in the h-ICH group (Fig 1C and 1E). Luxol fast blue/Cresyl violet staining revealed well-defined lesions and hemispheric enlargement in both groups. Quantification showed significantly larger injury volume and hemispheric enlargement in the c-ICH and h-ICH groups than in the respective sham groups (n = 5 mice/group, *p*<0.01; Fig. 1D and 1F).

### Brain water content

**Figure 2:**
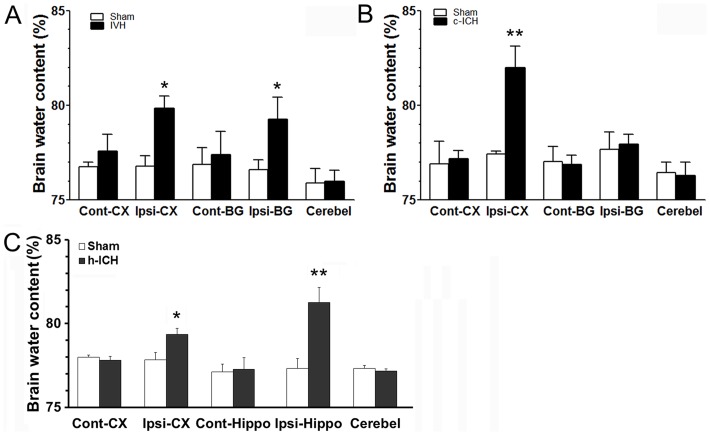
Brain edema is increased after intracerebral hemorrhage (ICH) in ventricle, cortex, and hippocampus. Three days after ICH, percentage brain water content was increased in the ipsilateral cortex and basal ganglia in the IVH group (n = 4 mice per group, A), in the ipsilateral cortex in the c-ICH group (n = 4 mice per group, B), and in the ipsilateral cortex and hippocampus in the h-ICH group (n = 4 mice per group, C). Cont-CX, contralateral cortex; Ipsi-CX, ipsilateral cortex; Cont-BG, contralateral basal ganglia; Ipsi-BG, ipsilateral basal ganglia; Cont-Hippo, contralateral hippocampus; Ipsi-Hippo, ipsilateral hippocampus; Cerebel, cerebellum. Values are means ± SD; *p<0.05, **p<0.01, t-test.

Brain edema is an important clinical complication of ICH. Compared with that in sham groups, brain water content increased in the ipsilateral basal ganglia and cortex of the IVH group (n = 4 mice/group, *p*<0.05; Fig. 2A), in the ipsilateral cortex of the c-ICH group (n = 4 mice/group, *p*<0.01; Fig. 2B), and in the ipsilateral cortex and hippocampus of the h-ICH group (n = 4 mice/group, *p*<0.05; Fig. 2C).

### Neuroinflammation

**Figure 3:**
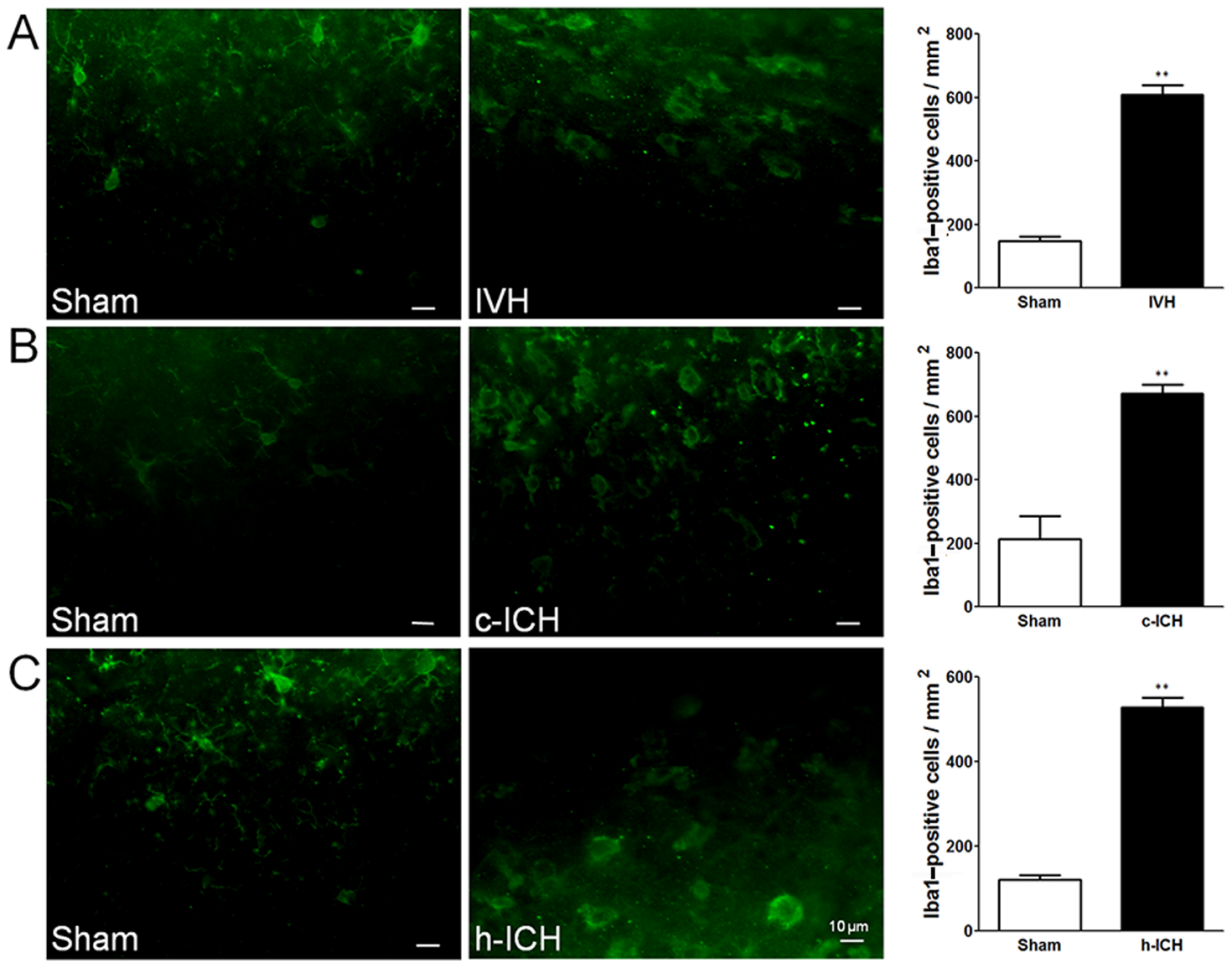
Microglia/macrophage activation is elevated 72 h after intracerebral hemorrhage (ICH) in ventricle, cortex, and hippocampus. Staining for Iba1 revealed activated microglia/macrophages in the brain regions around the lateral ventricles 72 h after intraventricular hemorrhage (IVH; A) and in the perihematomal regions of the frontal cortex (B) and hippocampus (C) 72 h after cerebral ICH (c-ICH) and hippocampal ICH (h-ICH), respectively. Quantification analysis showed that the number of activated microglia/macrophages in the IVH, c-ICH, and h-ICH groups was significantly greater than that in the respective sham groups (n = 5 mice per group). Scale bar  = 10 µm. Values are means ± SD; **p<0.01, t-test.

**Figure 4:**
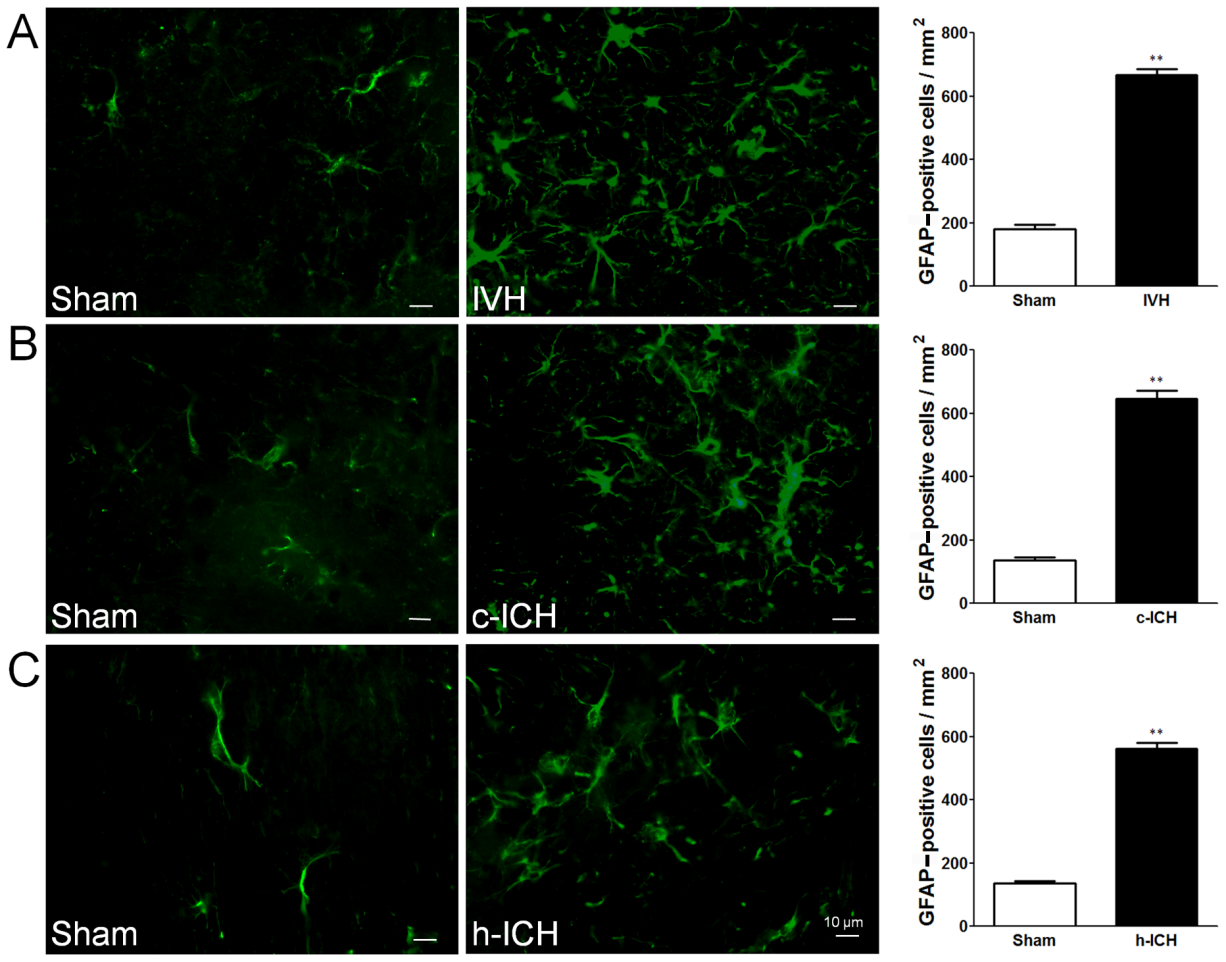
Astrocyte activation is elevated 72(ICH) in ventricle, cortex, and hippocampus. Staining for GFAP revealed reactive astrocytes in the brain regions around the lateral ventricles 72(IVH; A) and in the perihematomal regions of the frontal cortex (B) and hippocampus (C) 72 h after cerebral ICH (c-ICH) and hippocampal ICH (h-ICH), respectively. Quantification analysis showed that the number of reactive astrocytes in the IVH, c-ICH, and h-ICH groups was significantly greater than that in the respective sham groups (n = 5 mice per group). Scale bar  = 10 µm. Values are means ± SD; **p<0.01, t-test.

**Figure 5:**
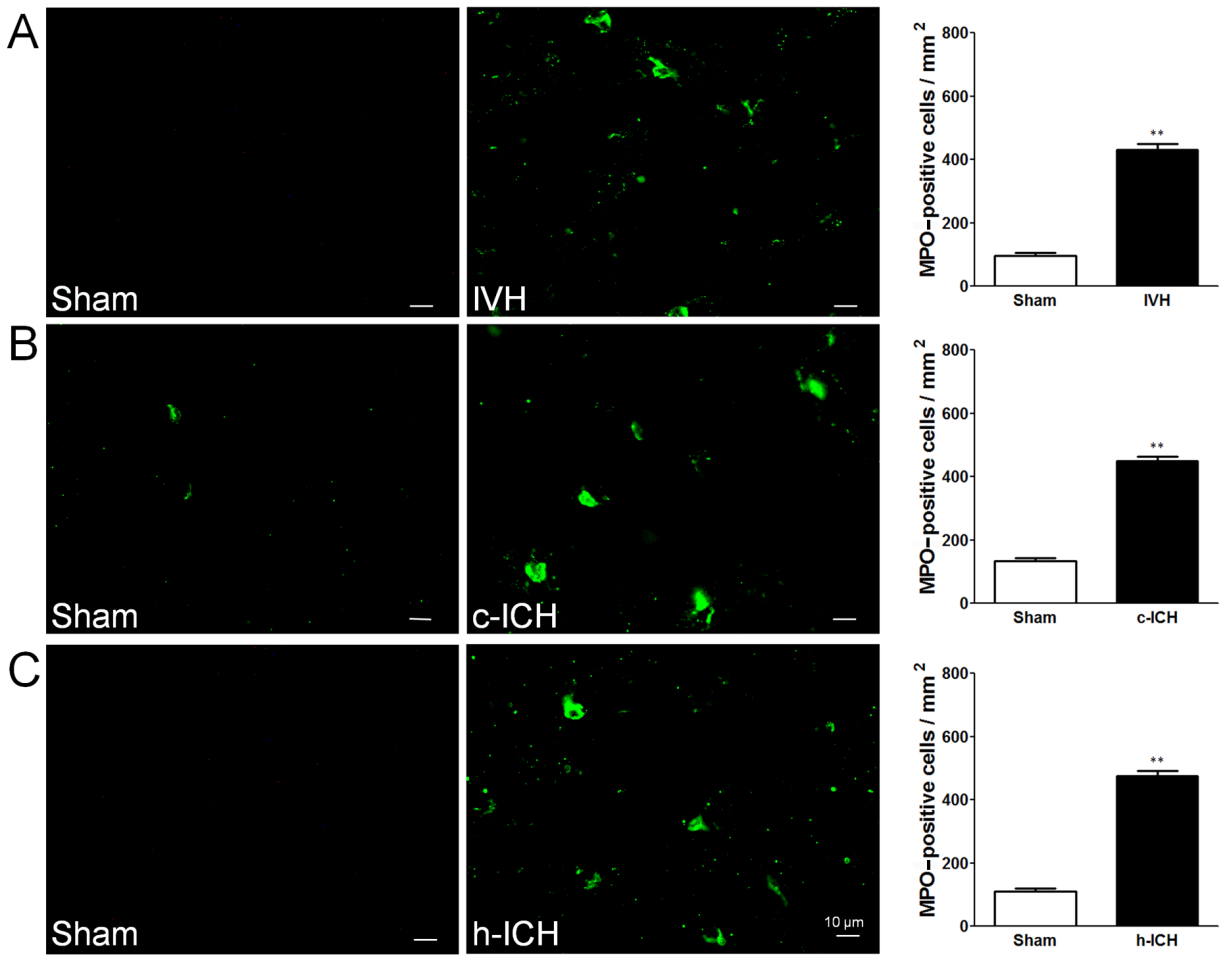
Neutrophil infiltration is elevated 72(ICH) in ventricle, cortex, and hippocampus. Staining for myeloperoxidase (MPO) revealed neutrophil infiltration in the brain regions around the lateral ventricles 72 h after intraventricular hemorrhage (IVH; A) and in the perihematomal regions of the frontal cortex (B) and hippocampus (C) 72 h after cerebral ICH (c-ICH) and hippocampal ICH (h-ICH), respectively. Quantification analysis showed that the number of MPO-positive cells in the IVH, c-ICH, and h-ICH groups was significantly greater than that in the respective sham groups (n = 5 mice per group). Scale bar  =  10 µm. Values are means ± SD; **p<0.01, t-test.

Early cellular inflammation contributes to secondary brain injury after ICH [4], [5]. We used morphologic criteria and a cell body diameter cutoff of 7.5 µm to define microglial/macrophage activation [37]. We used GFAP immunofluorescence labeling to examine the reactivity of astrocytes and MPO immunofluorescence labeling to examine the neutrophil infiltration [24]. At 72 h after ICH, activated microglia/macrophages (Fig. 3), reactive astrocytes (Fig. 4), and infiltrating neutrophils (Fig. 5) were evident in the brain regions around the lateral ventricles in the IVH mice and around the injury sites in the cortex and hippocampus in the c-ICH and h-ICH mice, respectively. Quantification analysis confirmed that significantly more activated microglia/macrophages, reactive astrocytes, and infiltrating neutrophils were present in the brains of mice that underwent the ICH models than in those of sham mice (n = 5 mice/group, all *p*<0.01; Fig. 3–5).

### Neuronal death

**Figure 6:**
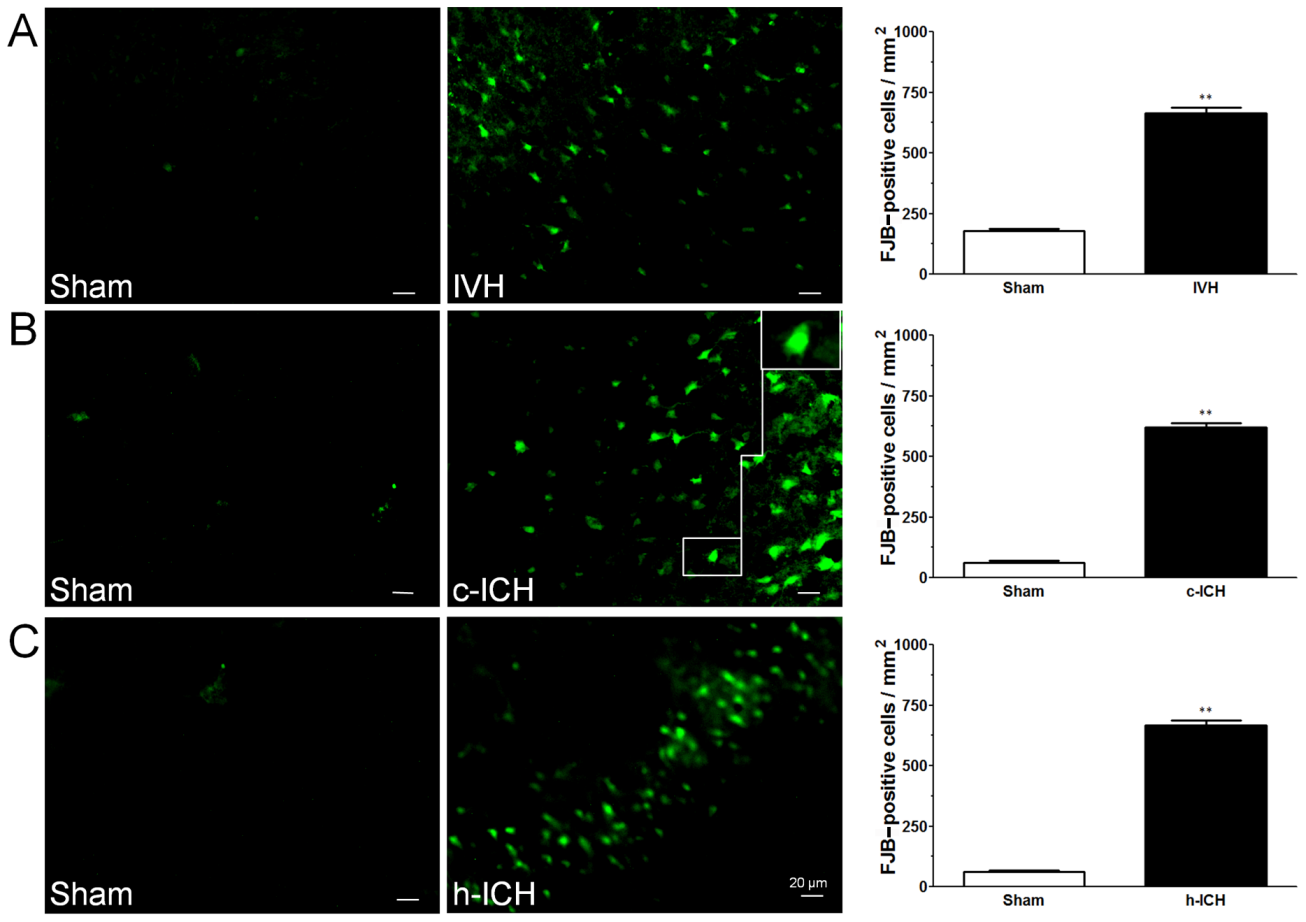
Neuronal degeneration is observed 72(ICH) in ventricle, cortex, and hippocampus. Fluoro-Jade B (FJB) staining was used to detect neuronal degeneration. FJB-positive cells were increased in the brain regions around the lateral ventricles 72 h after intraventricular hemorrhage (IVH; A) and in the perihematomal regions of the frontal cortex (B) and hippocampus (C) 72 h after cerebral ICH (c-ICH) and hippocampal ICH (h-ICH), respectively. Quantification analysis showed that the number of FJB-positive cells in the IVH, c-ICH, and h-ICH groups was significantly greater than that in the respective sham groups (n = 4 mice per group). Scale bar  =  20 µm. Values are means ± SD; **p<0.01, t-test.

FJB staining was used to detect neuronal degeneration. We observed more FJB-positive cells in the brain regions around the lateral ventricles of the IVH mice and around the injury sites in the cortex and hippocampus of the c-ICH and h-ICH mice, respectively, than in the corresponding sham groups (Fig. 6). Quantification analysis confirmed that significantly more FJB-positive cells were present in the brains of mice that underwent the ICH models than in those of sham mice (n = 4 mice/group, all *p*<0.01; Fig. 6).

### Changes in locomotor function

**Figure 7:**
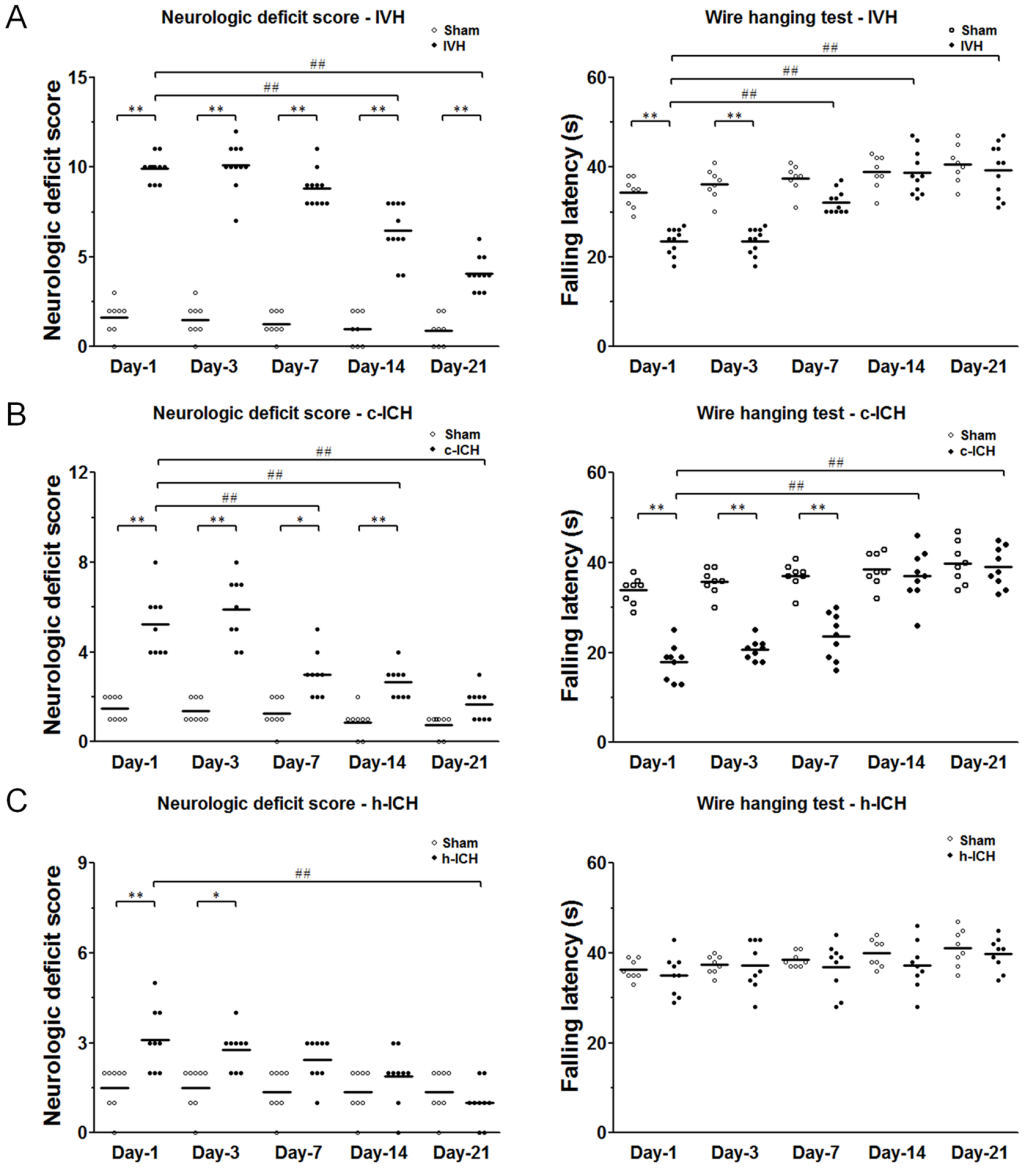
Changes in locomotor function after intracerebral hemorrhage (ICH) in ventricle, cortex, and hippocampus. Locomotor deficits were measured by a 24-point scoring system, in which higher scores indicate greater deficit, and by the wire-hanging test, which measures gripping and forelimb strength. (A) Mice in the IVH group had significantly greater motor deficit than did mice in the sham group at all time points after IVH (n = 11 mice per group, *F* = 138.3, all *p*<0.01) and showed gradual recovery from day 7 to day 21 post-ICH. Gripping and forelimb strength were significantly impaired in the IVH mice on days 1 and 3 after IVH (n = 11 mice per group, *F* = 27.72, both *p*<0.01). (B) Mice in the c-ICH group had significantly greater motor deficit than did mice in the sham group on days 1, 3, 7, and 14 after c-ICH (n = 9 mice per group, *F* = 35.31, *p*<0.05). Gripping and forelimb strength were significantly impaired in the c-ICH mice on days 1, 3, and 7 after c-ICH (n = 9 mice per group, *F* = 37.29, *p*<0.01). (C) Mice in the h-ICH group had significantly greater motor deficit than did mice in the sham group on days 1 and 3 post-ICH (n = 9 mice per group, *F* = 6.911, *p*<0.01 [day 1] and *p*<0.05 [day 3]). Gripping and forelimb strength were not significantly different between the h-ICH and sham groups at any time point studied (n = 9 mice per group, *F* = 1.803, all *p*>0.05). Values are means ± SD; *p<0.05, **p<0.01 vs. sham group; ^##^ *p*<0.01 vs. IVH, c-ICH, and h-ICH group on day 1; one-way or two-way ANOVA followed by the Bonferroni post hoc test.

Locomotor deficits were measured by a 24-point neurologic scoring system and the wire-hanging test on days 1, 3, 7, 14, and 21 after ICH. At all time points after ICH, deficit scores were significantly higher in IVH mice than in sham mice, indicting marked locomotor deficits (n = 11 mice/group, F = 138.3, *p*<0.01; Fig. 7A). The mice gradually recovered from days 7 to 21. In the wire-hanging test, IVH mice demonstrated marked deficits in gripping and forelimb strength compared with those of sham animals on days 1 and 3 post-ICH (n = 11 mice/group, F = 27.72, *p*<0.01; Fig. 7A). The mice exhibited some improvement on day 7 and complete recovery on days 14 and 21 post-ICH.

Mice in the c-ICH group had significantly higher neurologic deficit scores than did sham animals on days 1, 3, 7, and 14 post-ICH (n = 9 mice/group, F = 35.31, *p*<0.05; Fig. 7B). Locomotor deficit peaked on day 3, gradually recovered from day 7 to day 14, and returned to baseline on day 21. The wire-hanging test demonstrated marked deficits in gripping and forelimb strength compared with those of sham animals on days 1, 3, and 7 post-ICH (n = 9 mice/group, F = 37.29, *p*<0.01; Fig. 7B). Mice had completely recovered from these deficits on days 14 and 21 post-ICH.

Mice in the h-ICH group had significantly higher neurologic deficit scores than did sham animals on days 1 and 3 post-ICH (n = 9 mice/group, F = 6.911, *p*<0.05), but they recovered from day 7 to day 21. In the wire-hanging test, no difference was observed in gripping or forelimb strength between h-ICH mice and sham animals at any time point after ICH (n = 9 mice/group, F = 1.803, all *p*>0.05; Fig. 7C).

### Changes in emotion and memory function

**Figure 8:**
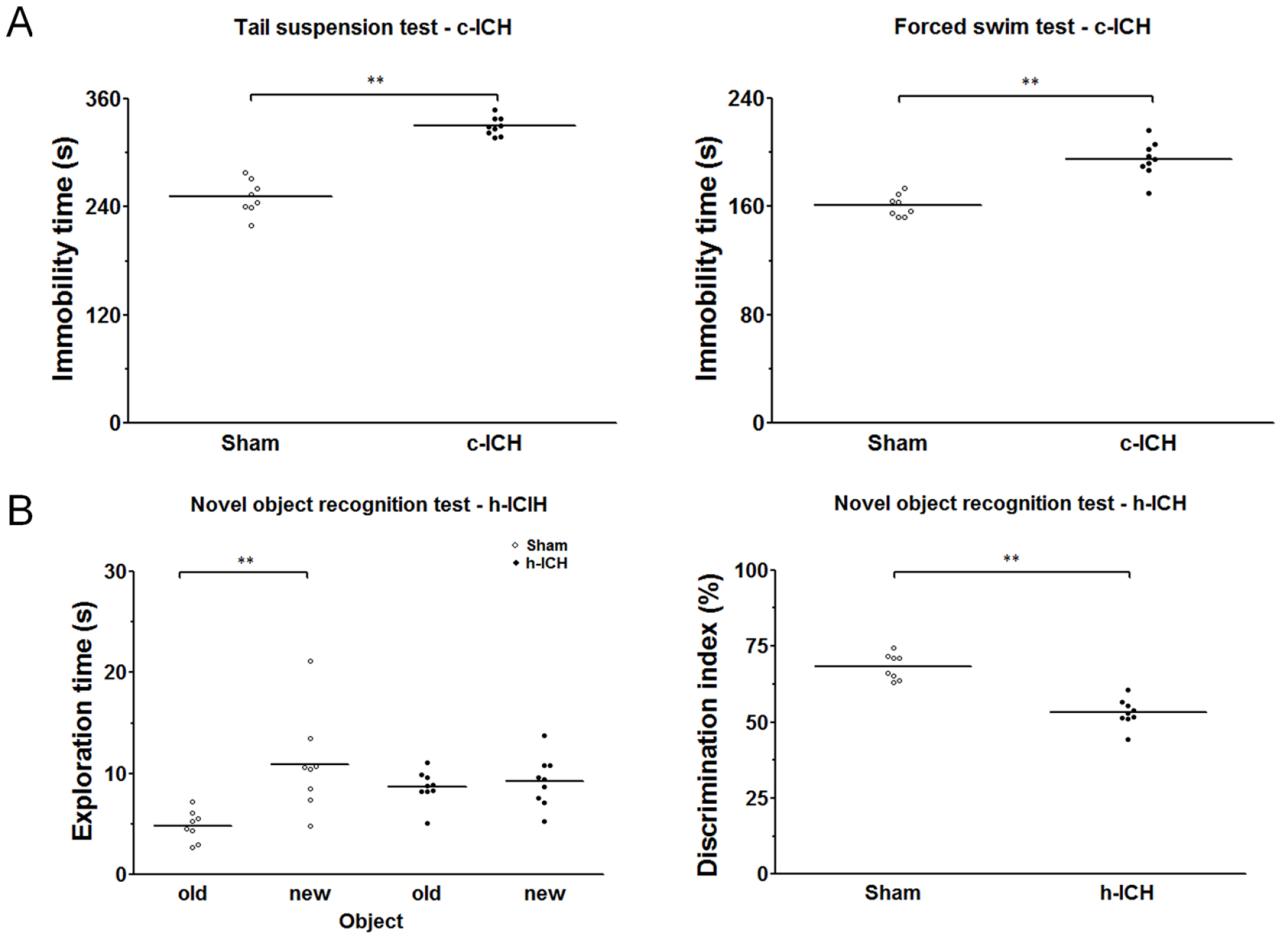
Changes in emotion and memory function after intracerebral hemorrhage (ICH) in cortex and hippocampus. (A) The immobility times in the tail suspension test and in the forced swim test were longer for mice subjected to cortical ICH than for sham-operated control mice (n = 9, *p*<0.01). (B) In the novel object recognition test, sham-operated mice spent more time exploring the novel object than the old object (n = 9 mice per group, *p*<0.01), but mice subjected to h-ICH spent a similar amount of time exploring the new and old objects (n = 9 mice per group, *p*>0.05). Additionally, the discrimination index in the h-ICH group was significantly smaller than that in the sham group (n = 9 mice per group, *p*<0.01). Values are means ± SD; **p<0.01, t-test.

Immobility times in the FST and TST were longer for c-ICH mice than for sham mice (n = 9 mice/group, *p*<0.01; Fig. 8A). In the novel object recognition test, sham animals spent more time exploring the novel object than the familiar object (n = 9 mice/group, *p*<0.01; Fig. 8B), but the h-ICH mice spent similar amounts of time exploring the new and the previously seen object (*p*>0.05). Additionally, the discrimination index was significantly smaller in the h-ICH group than in the sham group (n = 9 mice/group, both *p*<0.01; Fig. 8B).

## Discussion

A good animal model that can mimic the natural events of human ICH should have high standards, high reproducibility, and high clinical relevance. To achieve these goals, we used autologous whole blood or collagenase to establish IVH, c-ICH, and h-ICH models in mice. We validated and characterized these ICH models by histology, immunohistochemistry, and neurobehavioral tests. All three ICH models generated reproducible brain damage, brain edema, neuroinflammation, neuronal death, and consistent locomotor deficits. In addition, the c-ICH model produced emotional deficits and the h-ICH model produced cognitive deficits, closely mimicking the effects of human ICH in those brain regions.

To our knowledge, we are the first to establish the IVH and h-ICH models in mice. We have made efforts to mimic clinical ICH and optimize the models for accuracy, stability, and efficiency. For the blood IVH model, we omit anticoagulant in blood collection to avoid potential interference with the coagulation system [38]. In addition, we collect the blood onto Parafilm laboratory film to prevent blood clotting and reduce the total time for establishing this model. Additionally, we use autologous blood rather than donor blood to reduce the possibility of donor-induced inflammatory reaction and induction of the complement system [39]. In preliminary tests, we infused a variety of blood volumes (10, 25, and 40 µL). The 10-μL volume did not produce a large enough intraventricular hematoma, and 40 µL caused high mortality. The 25-μL infusion was able to cause sufficient blood accumulation and ventricular enlargement without high mortality. To ensure that the blood is infused into ventricle only, we recommend repeatedly checking the coordinates of the bregma, lambda, and entry point and ensuring that the mouse's head is exactly horizontal. If the needle is correctly positioned in the ventricle, there should be no blood backflow along the needle track. Blood accumulation in and enlargement of the bilateral ventricles on days 1 and 3 post-ICH confirmed successful establishment of the IVH model. Both 24-point neurologic deficit scoring and the wire-hanging test demonstrated marked and reliable locomotor deficits at early time points (days 1 and 3) and gradual recovery at later time points (days 7–21), suggesting that functional deficits and recovery also occurred in the IVH model.

For the collagenase-induced ICH models (c-ICH and h-ICH), we injected collagenase into two sites of the right frontal cortex to cause an expanded injury (primary motor and somatosensory area and secondary motor area) as described in a published protocol [21]. We used collagenase VII-S rather than collagenase IV because the latter contains nonspecific proteases that may cause unexpected brain damage. We chose a small volume of collagenase to restrict hematoma formation to the desired brain regions and to prevent backflow along the needle track, a common problem in most blood ICH models. In preliminary studies, we injected doses of collagenase (150 U/mL) into the right frontal cortex that ranged from 0.3 to 0.6 µL/site. However, only the 0.4-μL dose caused a controllable and reproducible hematoma, neither too small in the cortex nor too big beyond the cortex. For the h-ICH model, we injected 0.2 µL of collagenase into the right hippocampus according to the published coordinates [25]. In preliminary experiments, we tried injections of 0.3 µL and 0.1 µL collagenase (150 U/ml). However, the hematoma produced by 0.3 µL collagenase expanded out of the hippocampus, and the hematoma produced by 0.1 µL collagenase produced little damage.

Experimental evidence supports the premise that activated microglia/macrophages, reactive astrocytes, and infiltrating neutrophils could be major sources of proinflammatory mediators that contribute to secondary brain injury after ICH [4], [5]. Consistent with this notion, we previously reported that inhibition of microglial activation before or early after ICH decreases neuronal death and brain injury and improves functional outcome [14], [40]. Others have reported that neutrophil depletion reduces blood-brain barrier disruption, axon injury, and inflammation after ICH [41]. Reactive astrocytes may play diverse roles after brain injury such as regulating matrix metalloproteinase expression and activity, promoting the secretion of neurotrophic factors, and modulating microglial reactive oxygen species production [4], [5]. In this study, we showed for the first time that activated microglia/macrophages, reactive astrocytes, and infiltrating neutrophils accumulate around the ventricles in the IVH model and in the perihematomal regions in the c-ICH and h-ICH models 72 h after ICH, suggesting that cellular inflammation also contributes to secondary brain injury in these models.

The cortex contains primary motor and somatosensory areas and the secondary motor area. It has been shown that cortical injury induced by brain trauma causes motor and emotional deficits and that the emotional effects are not dependent on injury severity [42]. Our tests of locomotor function confirmed marked locomotor deficits early after c-ICH followed by gradual recovery. Moreover, the increase in immobility time of mice in the TST and FST indicates that the c-ICH model produced depression-like behavior. The fully recovered motor function at day 21 in this model suggests that the depression-like behavior was not caused by motor deficits. The TST and FST are well-established tests used to evaluate depression-like behavior in mice [32], [34], [43], but they have not been applied to ICH models. Our results demonstrate that these tests can be used to study emotional deficits after ICH.

The hippocampus plays an important role in the formation of new memories, and the novel object recognition task is used to evaluate recognition memory in rodents [36], [44]. In the h-ICH model, we found that mice had marked locomotor deficits at days 1 and 3 after ICH based on the neurologic score testing, but no deficits in the wire-hanging test, suggesting that, while h-ICH causes general locomotor deficits early, it does not cause deficits in gripping and forelimb strength, or at least not deficits severe enough to be detected. In contrast, mice that underwent h-ICH did exhibit impaired memory in the novel object recognition test, confirming damage to hippocampal memory formation. The deficits in the novel recognition test were not associated with motor function deficits because the test was conducted on day 21 after h-ICH, when motor function had recovered to baseline.

Our study does have some limitations. First, the aged population is most vulnerable to ICH, and aging exacerbates ICH-induced brain damage and functional deficits [11], [12]. Therefore, we need to establish ICH models in aged animals to increase clinical relevance. Second, we used a limited number of behavioral tests. Additional cognitive tests, such as the Morris water maze, and emotional tests, such as the sucrose preference test, should be assessed in our established ICH models. Third, we used collagenase in this study to induce ICH in the cortex and hippocampus and blood infusion to induce IVH. Although collagenase-induced ICH was previously thought to initiate greater neuroinflammation than blood-induced ICH [4], [12], in vitro and in vivo studies have argued against this notion. In vitro studies have shown that collagenase alone does not activate microglia [14], increase prostaglandin E2 production [45], or induce neuronal death [46]. Similarly, in vivo studies have shown that inflammation is more exaggerated in the blood ICH model than in the collagenase ICH model as a result of hemoglobin crystallization [47] and that the blood ICH model induces more pronounced alterations in inflammatory cytokine markers, despite equal ICH volumes [48]. Lastly, although rodents are used most often to study ICH, they are fundamentally different from humans in that they have less white matter and a lower glia-to-neuron ratio [4].

In conclusion, we have established and validated the IVH, c-ICH, and h-ICH models in mice. These three models will be of interest to neurologists and other scientists who conduct basic and translational stroke research because they closely mimic human ICH pathology by generating reproducible ventricular enlargement; hematoma volume; brain edema; neuroinflammation; neuronal death; and motor, emotional, and cognitive deficits. These mouse ICH models can be applied to investigate the pathophysiology of ICH and evaluate new therapeutic strategies for ICH.

## Supporting Information

Text S1 **Supporting text.**
(DOC)
