# Correction: Mouse Models of Intracerebral Hemorrhage in Ventricle, Cortex, and Hippocampus by Injections of Autologous Blood or Collagenase

**DOI:** 10.1371/journal.pone.0261640

**Published:** 2021-12-15

**Authors:** Wei Zhu, Yufeng Gao, Che-Feng Chang, Jie-ru Wan, Shan-shan Zhu, Jian Wang

The images for Figs 5A Sham, 5C Sham, 6B Sham, and 6C Sham are incorrect. The authors have provided the corrected Figs 5 and 6 below. The authors would like to apologize for any inconvenience caused.

**Fig 5:**
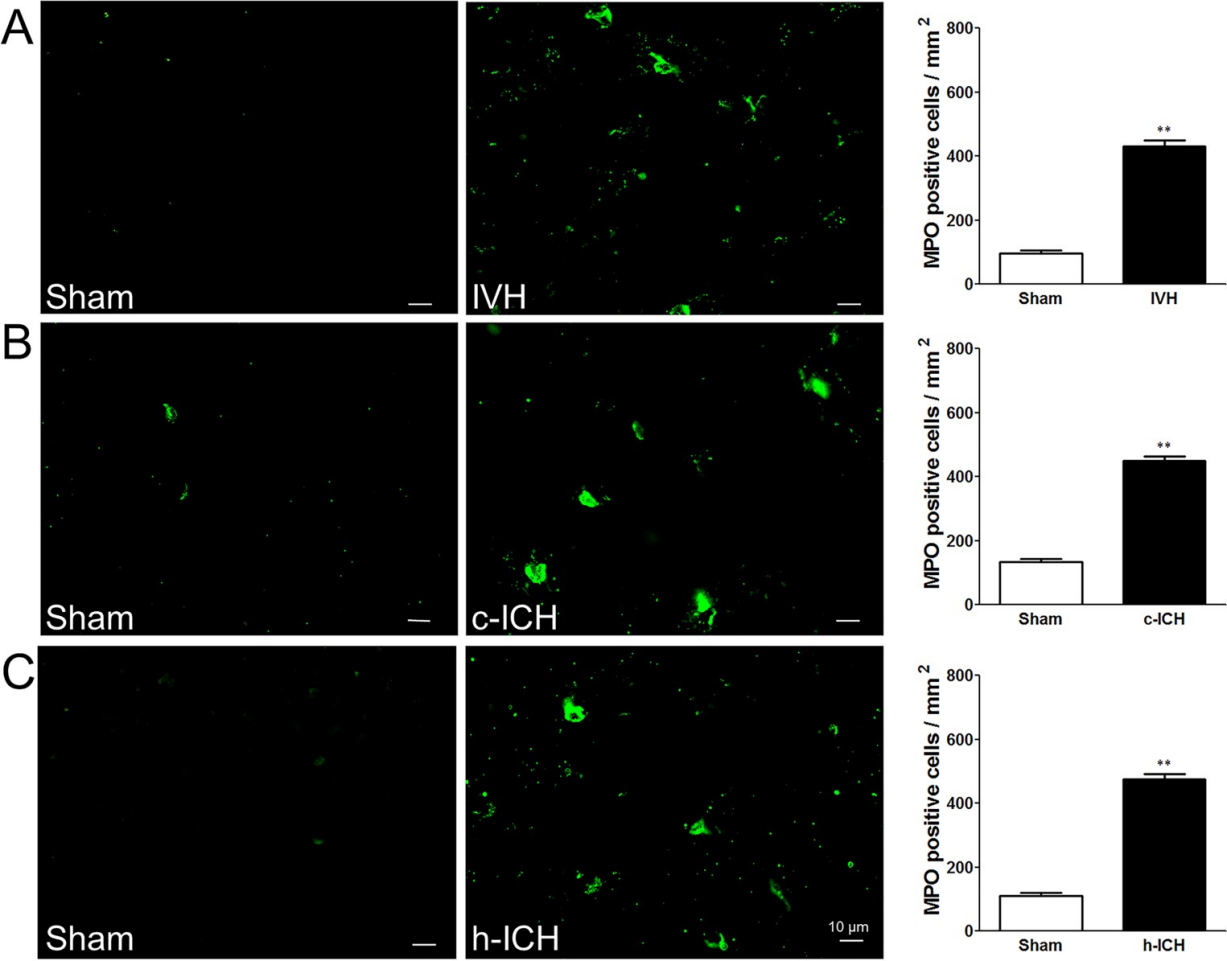
Neutrophil infiltration is elevated 72(ICH) in ventricle, cortex, and hippocampus. Staining for myeloperoxidase (MPO) revealed neutrophil infiltration in the brain regions around the lateral ventricles 72 h after intraventricular hemorrhage (IVH; A) and in the perihematomal regions of the frontal cortex (B) and hippocampus (C) 72 h after cerebral ICH (c-ICH) and hippocampal ICH (h-ICH), respectively. Quantification analysis showed that the number of MPO-positive cells in the IVH, c-ICH, and h-ICH groups was significantly greater than that in the respective sham groups (n  =  5 mice per group). Scale bar   =   10 μm. Values are means ± SD; **p<0.01, t-test.

**Fig 6:**
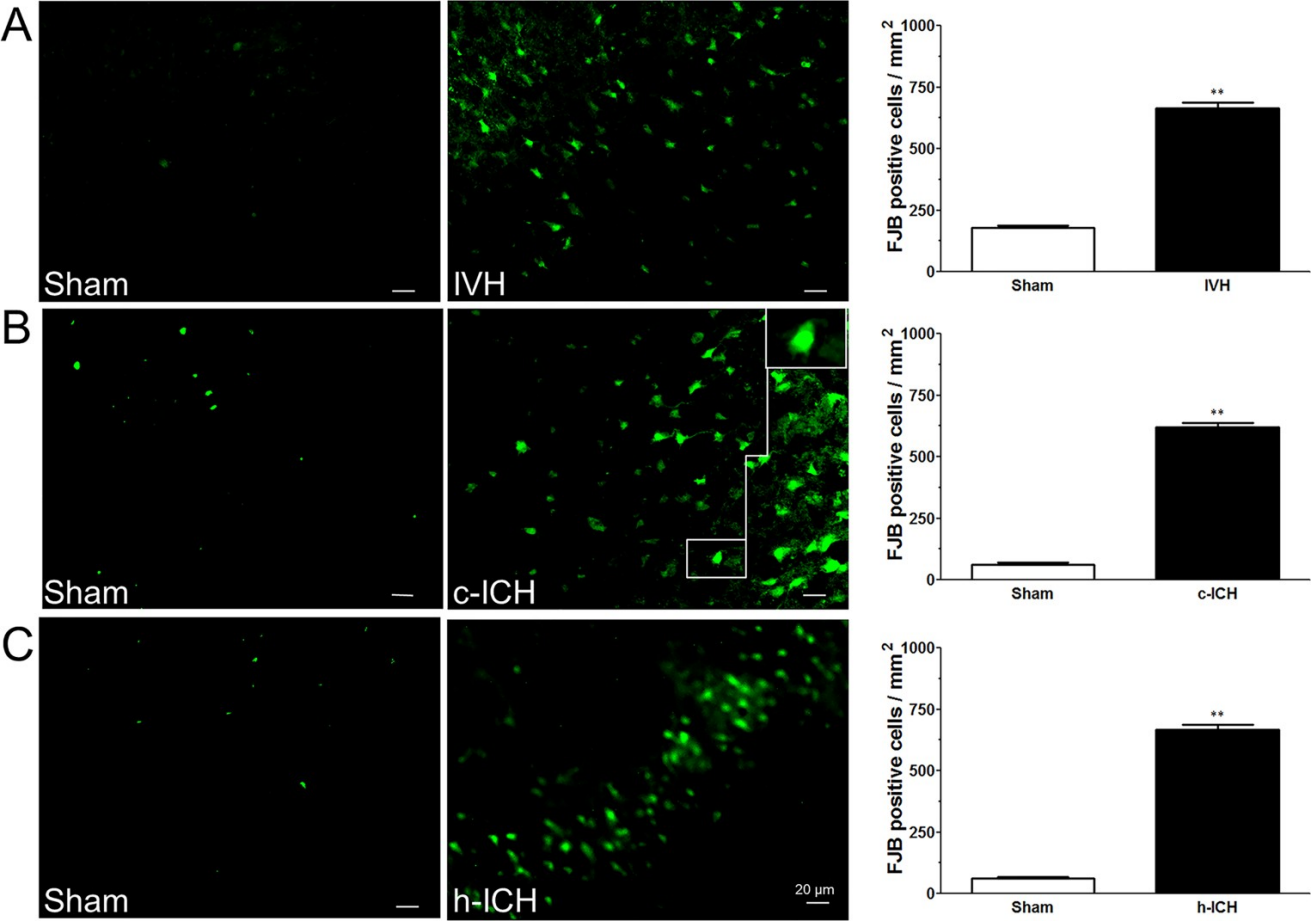
Neuronal degeneration is observed 72(ICH) in ventricle, cortex, and hippocampus. Fluoro-Jade B (FJB) staining was used to detect neuronal degeneration. FJB-positive cells were increased in the brain regions around the lateral ventricles 72 h after intraventricular hemorrhage (IVH; A) and in the perihematomal regions of the frontal cortex (B) and hippocampus (C) 72 h after cerebral ICH (c-ICH) and hippocampal ICH (h-ICH), respectively. Quantification analysis showed that the number of FJB-positive cells in the IVH, c-ICH, and h-ICH groups was significantly greater than that in the respective sham groups (n  =  4 mice per group). Scale bar   =   20 μm. Values are means ± SD; **p<0.01, t-test.

There are also errors in the Funding statement. The authors clarify that this work was not supported by the NIH grants. The correct Funding statement is as follows: This work was supported by a Tongji Hospital training grant and AHA 13GRNT15730001. The funders had no role in study design, data collection and analysis, decision to publish, or preparation of the manuscript.
