# Supplementary material for: Mouse Models of Intracerebral Hemorrhage in Ventricle, Cortex, and Hippocampus by Injections of Autologous Blood or Collagenase
Source: PLoS One. 2014 May 15;9(5):e97423. doi: 10.1371/journal.pone.0097423 (PMC4022524; doi:10.1371/journal.pone.0097423)
Supplement: Text S1 — Supporting text. (DOC) [file pone.0097423.s001.doc]

Supporting Information Text S1

**Protocols for mouse models of intraventricular hemorrhage (IVH),**

**cortical hemorrhage (c-ICH), and hippocampal hemorrhage (h-ICH)**

**Reagents and equipment**

1. Experimental animals: C57BL/6 male mice weighing 20–25 g (Experiment must be conducted in accordance with the national and institutional guidelines for the use of experimental animals).
2. Collagenase VII-S (sterile-filtered, high purity, purified by chromatography, Sigma-Aldrich Co., St Louis, MO, USA).
3. Isoflurane (Baxter Healthcare Co., Deerfield, IL, USA) (Isoflurane is a halogenated ether used for inhalational anesthesia. Sealing equipment is needed to preserve it).
4. Sodium chloride injection (Hospira Inc., Lake Forest, IL, USA).
5. Povidone-iodine prep pads (Dynarex Co., Orangeburg, NY, USA).
6. 70% Alcohol (MediChoice, Hanover, MD, USA).
7. Cyanoacrylate glue (Henkel Consumer Adhesive Inc., Avon, OH, USA).
8. Bone wax (Ethicon, Somerville, NJ, USA).
9. Stereotaxic instrument (Stoelting Co., Wood Dale, IL, USA).
10. Digital lab standard with LED digital display (Stoelting Co., Wood Dale, IL, USA).
11. Quintessential stereotaxic injector (Stoelting Co., Wood Dale, IL, USA).
12. MI-150 High intensity illuminator (Dolan-Jenner Industries, Boxborough, MA, USA).
13. FHC DC Temperature Controller (FHC Inc., Bowdoin, ME, USA).
14. Microdrill, 1 mm- (for IVH model) and 0.9 mm- (for c-ICH and h-ICH models) drill bits (Foredom Electric Co., Bethel, CT, USA).
15. Anesthesia vaporizer (Matrx Medical Inc., Minneapolis, MN, USA).
16. Air/oxygen mixer flowmeter (Fraser Sweatman Inc., Hatfield, PA, USA).
17. Hamilton syringes, 1 μL and 50 μL (Hamilton Co., Reno, NV, USA).
18. Gauze sponges (Covidien Co., New Haven, CT, USA).
19. Parafilm M laboratory wrapping film (Pechiney Plastic Packaging Co., Chicago, IL, USA).
20. Alcohol prep pads (Covidien Co., New Haven, CT, USA).
21. Surgical mask (Kimberly-Clark Co., Irving, TX, USA).
22. Latex gloves (Kimberly-Clark Co., Irving, TX, USA).
23. Marker pen (Sharpie, Baltimore, MD, USA).
24. Sterile, stainless steel surgical blades (Sklar Instruments, West Chester, PA, USA).
25. Surgical instruments: dissection scissors, straight surgical forceps.

**Procedure**

**IVH model with autologous whole blood**

***Preoperative setup***

1. Set up the stereotactic apparatus, stereotactic injector, microdrill, high intensity illuminator, and temperature controller (Figure S1A). Adjust the oxygen flow to 0.2 L/min and air flow to 0.8 L/min.
2. Weigh and record the body weight of the mouse. Choose mice that weigh between 20 and 25 g.
3. Put mouse into a clean induction chamber and anesthetize it with isoflurane (3–4% for induction and 1–2% for maintenance) evaporated in an oxygen-air mixture (20%:80%) until the mouse has lost consciousness. Ensure that the chamber is secured.

When anesthetizing the mouse with isoflurane, depth of anesthesia should be carefully monitored by assessing respiration rate and pedal withdrawal reflexes.

1. Shave the scalp with an electric shaver. Place the mouse onto a gas anesthesia platform and carefully secure the mouse’s head onto the stereotactic apparatus using two ear bars. Snugly attach a nose cone and adjust the isoflurane concentration to 1–2%. Maintain the oxygen flow and air flow unchanged.

The mouse’s head should be secured; its upper surface should be positioned horizontally to the base of the stereotactic frame. The nose cone should be well-fitted to the mouse’s nose for isoflurane inhalation.

1. Monitor the mouse’s rectal temperature with a thermometer. A temperature controller should be used to maintain rectal temperature at 37 ± 0.5°C throughout the entire operational procedure.
2. Apply ophthalmic antibiotic cream to both eyes.
3. Disinfect the surgical field of the head with povidone iodine pads, and rinse with 70% ethanol. Repeat alternating applications of povidone iodine pads and 70% ethanol pads three times.

***Exposure***

1. Make a 1-cm-long midline incision in the scalp beginning midway between the eyes, and terminate behind the lambda.
2. Use a cotton swab to clear away the soft tissue that covers the skull. The sagittal suture, coronal suture, bregma, and lambda should be clearly identified. Mark the bregma and lambda with a marker pen (Figure S1B).
3. Mount the Hamilton syringe onto the injection pump and then adjust the stereotactic manipulator arms to position the needle directly over the bregma. Reset the coordinates to zero (x, y, and z axes). Adjust the stereotactic manipulator arms again to position the needle directly over the lambda. The x and z axis coordinates of the lambda should be the same as the coordinates of the bregma, but the y coordinate should be different from that of the bregma. The difference in value is the vertical distance from bregma to lambda. If the x and z axis coordinates of lambda are not the same as those of the bregma, adjust the ear bars up or down, left or right to ensure accurate positioning.
4. Adjust the stereotactic manipulator arms to position the needle directly over the bregma again (x, y, and z axis coordinates should be zero). Adjust the stereotactic manipulator arms to position (0.5 mm posterior and 1.0 mm lateral of the bregma to the right). Mark the entry point on the skull.
5. Drill a small cranial burr hole through the skull at the marked entry point; make sure not to damage the dura mater.
6. Recheck and verify the coordinates of the bregma, lambda, and entry point.

***Autologous whole blood injection***

1. Disinfect the tail skin with 70% alcohol and then immerse the mouse’s tail in warm, sterile water (40°C) for 2 min. Immersing the tail in warm water dilates the central artery and is essential to acquire enough blood (Figure S2). Do not scrub the tail, as this will result in leukocytosis and increase the risk of tissue fluid contamination.
2. Use a sterile needle (25 G) to puncture the central tail artery. Then collect blood drops onto a piece of Parafilm laboratory film.
3. Transfer 25 μL of blood quickly from the Parafilm laboratory film into the Hamilton syringe. Avoid trapping any air bubbles while drawing the blood into the syringe. This procedure should be performed quickly (<1 min) to prevent blood clotting. We use Parafilm laboratory film to collect the blood because it lacks adhesive properties. The blood on the film gathers together because of high surface tension.
4. Reattach the Hamilton syringe onto the injection pump. Adjust the stereotactic manipulator arms to position the needle directly over the entry point and slowly insert into the right ventricle 2.5 mm below the surface of the skull.
5. Inject 25 μL of autologous blood at a rate of 5 μL • min^-1^. For control groups, we infuse an equal amount of saline.
6. During injection, carefully watch whether blood flows back along the needle track. If the needle is in the ventricle, the injected blood will not flow back at this rate. If the needle is not in the ventricle, the blood will flow back because of the high intracranial pressure and resistance caused by the blood injected into the brain tissue.
7. After blood infusion, the needle is left in place for 10 min.
8. Slowly remove the Hamilton syringe from the target point at a rate of 1 mm • min^-1^.
9. Withdraw the needle slowly to avoid backflow of the injected blood along the needle track.

***Closure and postoperative care***

1. Seal the burr hole with bone wax.
2. Close the scalp incision with cyanoacrylate tissue glue.
3. Remove the mouse from the stereotactic apparatus and place it in an individual cage. For those mice that are unable to move, provide a nutrient gel on the bottom of the cage. Mice that lose more than 20% of their body weight on the first day should be excluded.

**c-ICH and h-ICH models with collagenase injection**

***Preoperative setup***

Same as steps 1–7 of the IVH model.

***Exposure***

1–3. Same as steps 1–3 of the IVH model.

4. Adjust the stereotactic manipulator arms to position the needle directly over the bregma (x, y, and z axis coordinates should be zero). For the c-ICH model, adjust the stereotactic manipulator arms to the 1^st^ position (0.0 mm anterior and 1.5 mm lateral of the bregma to the right) and 2^nd^ position (1.0 mm anterior and 2.0 mm lateral of the bregma to the right). Mark the entry points on the skull. For the h-ICH model, adjust the stereotactic manipulator arms to the position 2.5 mm posterior and 1.7 mm lateral of the bregma to the right. Mark the entry point on the skull.

5–6. Same as steps 5–6 of the IVH model.

***Collagenase injection***

1. Fill a 1-μL Hamilton syringe with 0.4 μL or 0.2 μL of collagenase (150 U • ml^-1^). We inject 0.4 μL of collagenase into each site of the right frontal cortex for the c-ICH model and 0.2 μL of collagenase into the CA1 region of the right hippocampus for the h-ICH model.

2. Adjust the stereotactic manipulator arms to position the needle directly over the entry point and slowly insert it into the right cortex to a depth of 1.0 mm below the surface of the skull or into the right hippocampus to a depth of 1.8 mm below the surface of the skull. Inject the collagenase at a rate of 0.1 μL • min^-1^. For the c-ICH model, leave the needle in position for 20 min after the first injection and then do the second injection. For control groups, we infuse equal amounts of saline.

3. After injection, the needle is left in position for 20 min in the c-ICH model and for 10 min in the h-ICH model.

4. Slowly remove the Hamilton syringe from the target point at a rate of 1 mm • min^-1^.

Withdraw the needle slowly to avoid backflow of the injected collagenase along the needle track.

***Closure and postoperative care***

Same as steps 1–3 of the IVH model.

Diagrams show collagenase-induced hematomas in the cortex after the c-ICH model (Figure S3A) and after the h-ICH model (Figure S3B).

**Figure S1** (A) The setup of the stereotactic apparatus and other devices used in the ICH models. A, stereotactic injector; B, stereotactic instrument (including gas anesthesia platform, mouse mask, and stereotactic frame); C, digital lab standard with LED digital display; D, microdrill; E, temperature controller; F, high-intensity illuminator. (B) Landmarks of mouse skull on a mouse mounted in the stereotactic frame. A, bregma; B, lambda; C, coronal suture; D, sagittal suture.

**
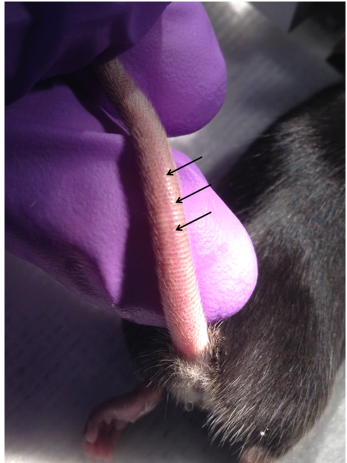
**

**Figure S2** Dilated central tail artery of a mouse. The mouse tail was immersed in warm water (40°C) for 2 min. The tail tip is held tightly for blood collection by arterial puncture. Arrows: mouse central tail artery.

**
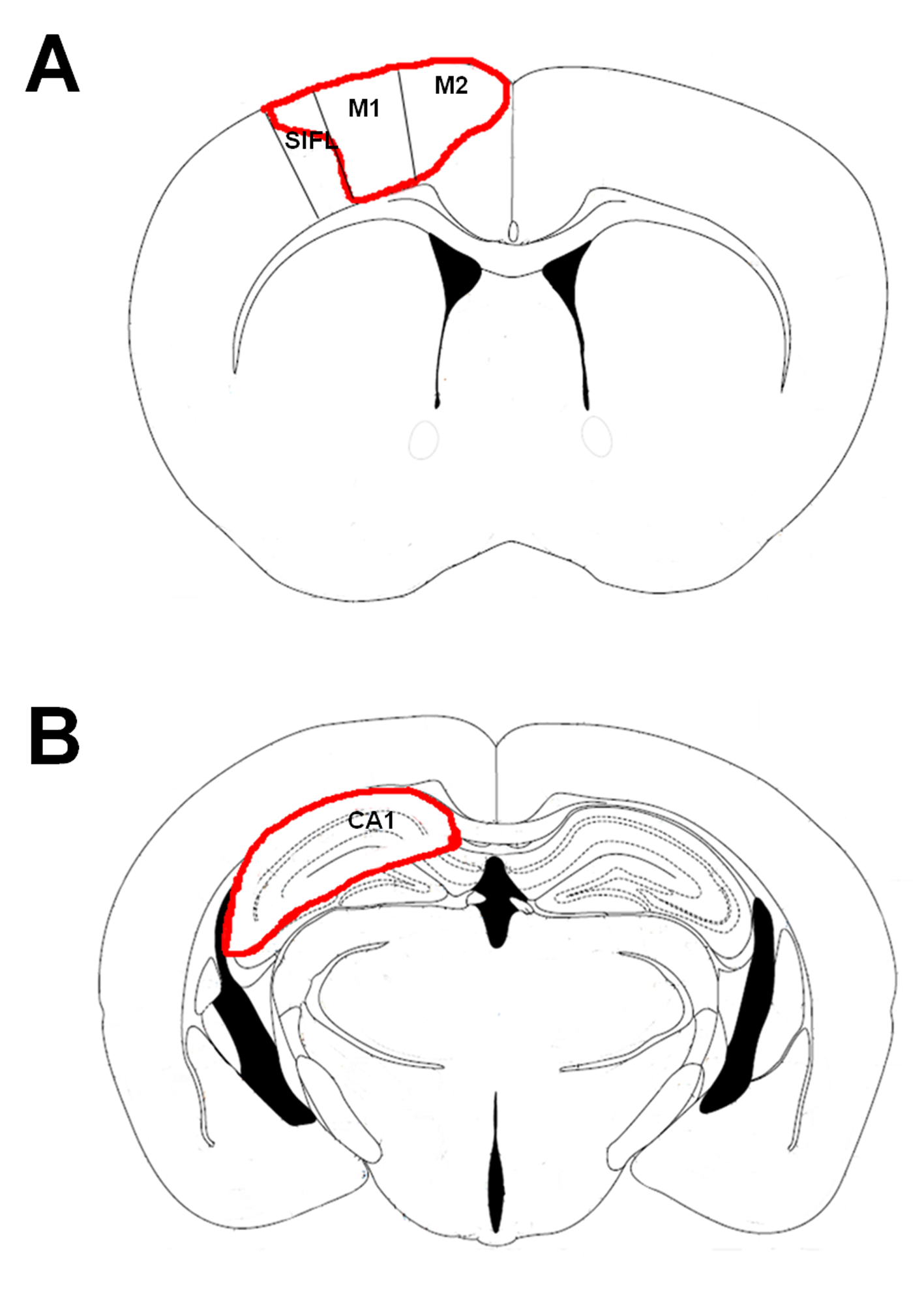
**

**Figure S3** Diagrams showing hematomas induced by collagenase in the cortex after the cortical intracerebral hemorrhage (c-ICH) model (A) and in the hippocampus after the hippocampal intracerebral hemorrhage (h-ICH) model (B). In the c-ICH model, hematoma affects primary motor cortex (M1), secondary motor cortex (M2), and primary somatosensory fore-limb (SIFL). In the h-ICH model, hematoma affects the CA1-3 regions of the hippocampus.
